# Increased Caspase-6 activity in the human anterior olfactory nuclei of the olfactory bulb is associated with cognitive impairment

**DOI:** 10.1186/s40478-016-0400-x

**Published:** 2016-12-08

**Authors:** Benedicte Foveau, Steffen Albrecht, David A. Bennett, José A. Correa, Andrea C. LeBlanc

**Affiliations:** 1 Bloomfield Center for Research in Aging, Lady Davis Institute for Medical Research, Jewish General Hospital, 3755 ch. Côte Ste-Catherine, Montreal, QC Canada; 2 Department of Pathology, Montreal Children’s Hospital and McGill University, Montreal, QC Canada; 3 Rush Alzheimer’s Disease Center, Rush University Medical Center, Chicago, IL USA; 4 Department of Mathematics and Statistics, McGill University, Montreal, QC Canada; 5 Department of Neurology and Neurosurgery, McGill University, Montreal, QC Canada

**Keywords:** Alzheimer Disease, Caspase-6, Olfactory bulb, Entorhinal cortex, Hippocampus, CA1, Memory scores, Cognitive scores, Neurofibrillary tangles, Amyloid beta peptide, Mild cognitive impairment, Non-cognitively impaired

## Abstract

Abnormally elevated hippocampal Caspase-6 (Casp6) activity is intimately associated with age-related cognitive impairment in humans and in mice. In humans, these high levels of Casp6 activity are initially localized in the entorhinal cortex, the area of the brain first affected by the formation of neurofibrillary tangles, according to Braak staging. The reason for the high vulnerability of entorhinal cortex neurons to neurofibrillary tangle pathology and Casp6 activity is unknown. Casp6 activity is involved in axonal degeneration, therefore, one possibility to explain increased vulnerability of the entorhinal cortex neurons would be that the afferent neurons of the olfactory bulb, some of which project their axons to the entorhinal cortex, are equally degenerating. To examine this possibility, we examined the presence of Casp6 activity, neurofibrillary tangle formation and amyloid deposition by immunohistochemistry with neoepitope antisera against the p20 subunit of active Casp6 and Tau cleaved by Casp6 (Tau∆Casp6), phosphorylated Tau paired helical filament (PHF-1) antibodies and anti-β-amyloid antiserum, respectively, in brains from individuals with no or mild cognitive impairment and Alzheimer disease (AD) dementia. Co-localization of Casp6 activity, PHF-1 and β-amyloid was detected mostly in the anterior olfactory nucleus (AON) of the olfactory bulb. The levels of active Casp6 in the AON, which were the highest in the AD brains, correlated with PHF-1 levels, but not with β-amyloid levels. AON Tau∆Casp6 levels correlated with entorhinal cortex Casp6 activity and PHF-1 levels. Multiple regression analyses demonstrated that AON Casp6 activity was associated with lower global cognitive function, mini mental state exam, episodic memory and semantic memory scores. These results suggest that AON Casp6 activity could lead to Casp6-mediated degeneration in the entorhinal cortex, but cannot exclude the possibilities that entorhinal cortex degeneration signals degeneration in the AON or that the pathologies occur in both regions independently. Nevertheless, AON Casp6 activity reflects that of the entorhinal cortex.

## Introduction

Identification of specific early molecular mechanisms underlying neurodegeneration and leading to age-related cognitive impairment and AD dementia may provide novel therapeutic targets against which efficient therapies can be developed. Our laboratory has focused on Casp6, a cytosolic cysteinyl protease, which is almost undetectable in normal aged human brain tissues [25], but extremely abundant and active in both sporadic [27] and familial [2] AD. From early to late stages of AD, Casp6 activity is detected in neurofibrillary tangles (NFT), neuritic plaques and neuropil threads, the main pathological lesions of AD [3, 27]. The activation of Casp6 in human primary neuron cultures increases amyloid beta peptide production [32, 36, 38]. Furthermore, Casp6 activity is implicated in age-related cognitive impairment. In aged humans, higher levels of Casp6 activity in the entorhinal cortex (ERC) and Cornus Ammonis 1 (CA1) regions of the hippocampus predict lower performance in episodic memory, the first type of memory to be decreased in AD [3, 47]. Post-natal expression of a self-activated form of human Casp6 in the hippocampal CA1 region of mouse brains induces age-related spatial and episodic memory impairment and is associated with early inflammation and neuronal loss [39]. Unlike the other two effector caspases, Casp3 and Casp7, Casp6 does not induce cell death when activated [26, 33]. However, Casp6 causes axonal degeneration. Casp6 proteolytically cleaves cytoskeletal proteins crucial to neuronal integrity and function, such as alpha-tubulin, microtubule-associated protein Tau and post-synaptic density proteins regulating the actin cytoskeleton of the dendritic spines in synapses [29, 33, 56]. Casp6 is activated and associated with axonal degeneration in nerve growth factor-deprived mouse sensory neuron cultures, coordinately with cellular apoptosis generated by effector Casp3 activation [45, 52, 53, 64]. In human primary neurons transfected to overexpress wild type or Swedish and London mutant amyloid precursor protein (APP), three conditions associated with familial AD [11, 48], Casp6 is activated in the absence of Casp3 activity and causes axonal degeneration [37, 55]. This is consistent with the absence of significant amounts of either active Casp3 or Casp7 in AD brains that exhibit high amounts of active Casp6 [37, 50, 57].

The early vulnerability of the ERC and CA1 to age-related Casp6 activation is commensurate with early NFT formation in pre-clinical Braak stages I and II of AD [12]. In humans, neurons from the ERC project their axons towards the CA1 region and receive input from the olfactory bulb neurons. Therefore, degenerating neurons of the olfactory bulb could cause degeneration of input neurons of the ERC, which require synaptic signals for survival. Specifically, mitral and tufted neurons of the olfactory bulb receive input from neurons of the olfactory epithelium where the olfactory nerves of the epithelium cross the cribriform plate of the ethmoid bone. Mitral and tufted neurons direct their axons to the olfactory tract and can form clusters of axons called the AON in the olfactory bulb and the olfactory tract, or project their axons to the piriform cortex, the olfactory tubercle and the lateral ERC [51].

The possibility that olfactory bulb degeneration is related to hippocampal degeneration is supported by the fact that odor identification impairment in aging is associated with lower cognition [67]. Furthermore, impaired odor identification predicts the progression of non-cognitively impaired (NCI) to mild cognitively impaired (MCI) and of MCI to AD, and is associated with decreased episodic and semantic memory as well as decreased perceptual speed [68]. Impaired odor identification is also associated with NFT pathology in the ERC [66]. Furthermore, the levels of Tau and amyloid pathology in the olfactory bulb echoes the presence and severity of AD brain pathologies found in other regions, suggesting that brain biopsies of the olfactory bulb could be used for early diagnosis or to monitor the effects of drug treatments [6]. Atrophy of the olfactory bulb is detected in MCI and in AD and correlate with lower mini mental state exam (MMSE) scores [59–61]. The AON is the area most affected by neuronal loss in AD [22]. Cholinergic neurons of the olfactory system decrease by one third [35, 54] and mitral cells are reduced significantly in AD olfactory bulb [28, 58]. The AON is also the area where NFT and senile plaques occur [16, 19, 30, 35, 41, 44, 46], and the number of NFT is compatible with a diagnosis of AD with an accuracy of 93.3% [34]. The NFT pathology of the AON correlates with Braak stages, Lewy body pathology and the Apo E4 genotype [5, 23, 63].

Given the projection of olfactory tracts to the ERC, here, we examined Casp6 activation in the olfactory bulb of NCI, MCI and AD individuals. Casp6 activity was mainly observed in the AON region of the olfactory bulb. Levels of Casp6 activity correlated with PHF-1 NFT pathology in the AON. Casp6 activity and PHF-1 NFT pathology in the AON correlated with the levels of Casp6 activity in the ERC. In all AD dementia cases and a few MCI cases, Casp6 activity in the AON region was higher than that observed in the ERC. A similar trend was observed for PHF-1. By contrast, levels of β-amyloid immunopositive pathology were generally similar in the AON and the ERC. Levels of AON Casp6 activity were associated with lower cognition in four independent cognitive measures. These results suggest that AON Casp6 activity could precede and induce Casp6-dependent ERC degeneration but do not exclude the possibility that ERC degeneration triggers AON degeneration. However, the results show an association between AON and ERC degeneration.

## Materials and methods

### Human brain tissues

Olfactory bulb and hippocampal tissue sections were obtained from the Religious Orders Study (ROS). The study was approved by the Institutional Review Board of Rush University Medical Center. All subjects signed informed consent and an Anatomic Gift Act. The McGill University Institutional Review Board approved the use of these human tissues in research. Details of the clinical and pathologic evaluation have been previously reported [9]. Yearly clinical evaluations included a medical history, neurological and cognitive assessment and a clinical diagnosis. Each case underwent MMSE to describe the cohort. Tests of visuospatial ability, perceptual speed and episodic, semantic and working memory evaluations yearly and these were converted to Z scores (using the mean and S.D. at baseline). A global cognitive score was developed by averaging the 19 cognitive function tests used to assess the five cognitive domains. Diagnostic classifications were provided by a clinician following the National Institute of Neurological and Communicative Disorders and Stroke and the AD and Related Disorders Association (NINCDS-ADRDA) criteria, without consideration of the pathological evaluation [42]. The diagnosis of AD dementia was assigned to cases with a history of cognitive decline and evidence of impairment of episodic memory and one other cognitive domain. MCI referred to cases of cognitive impairment without dementia. In the absence of dementia or MCI, cases were assigned a NCI diagnosis. At autopsy, brains underwent standard neuropathological evaluations, including Braak Stage determination. Neuropathological examination of the autopsied brains was done by a board-certified neuropathologist according to a modified Consortium to establish a registry for Alzheimer’s Disease (CERAD) criteria, without access to clinical information [8]. Brain tissue sections were stained with a Bielchowski silver stain to reveal neuritic and diffuse plaques and NFT. Braak stages were determined based on NFT severity and distribution [31]. Other pathologies were examined. Only 2 AD from all subjects examined had typical hippocampal sclerosis. Lewy body disease (alpha-synuclein), TDP-43, atheriosclerosis, cerebral amyloid angiopathy, cerebral artherosclerosis, gross chronic infarction and gross cerebral infarcts were present and mostly equivalent in the brain of NCI, MCI and AD individual. Chronic microinfarcts and micro infarctions were present only in NCI and AD cases. Note that the clinical diagnosis was not always in agreement with the neuropathological diagnosis [8] and that our designation of AD, MCI and NCI in this paper is based on the cognitive assessment.

A total of 34 cases comprising 18 NCI, 9 MCI and 7 AD dementia were studied. Table 1 shows the demographic and the clinical characteristics of the study group including cognitive scores taken within a year of death and the Braak stages of NFT pathology determined at autopsy. The number of years of education and post-mortem interval (PMI) were similar among the three groups. The AD cases were slightly older and had lower cognitive scores than the NCI and MCI groups.

**Table 1:** Demographics and clinical characteristics of the study groups

Characteristic	NCI (*n* = 18)	MCI (*n* = 9)	AD (*n* = 7)
Age at death (yrs)	86.3 (6.4)	84.9 (6.8)	92.4 (4.7)
Male : Female	6 : 12	5 : 4	2 : 5
Education (yrs)	17.8 (3.2)	18.0 (1.8)	17.9 (2.1)
Post mortem interval, median (IQR)	5.6 (5.3–6.3)	5.1 (4.5–7.3)	5.8 (4.5–13.3)
Apolipoprotein E ε4, n (%)	3 (16.7%)	2 (11.1%)	0
Mini mental state exam (MMSE)	28.3 (1.8)	26.7 (1.9)	14.4 (8.3)
Cognitive test scores			
Global Cognitive Scores	0.03 (0.4)	−0.4 (0.5)	−1.6 (0.8)
Episodic memory	0.4 (0.6)	−0.3 (0.9)	−2.2 (1.3)
Working memory	−0.02 (0.5)	−0.4 (0.8)	−0.8 (0.4)
Semantic memory	−0.1 (0.6)	−0.3 (0.6)	−1.3 (0.9)
Visuospatial activity	−0.1 (0.6)	−0.4 (0.9)	−1.2 (0.3)
Perceptual Speed	−0.3 (0.5)	−0.9 (0.7)	−1.6 (0.4)
Braak Stages, n (%)			
0	1 (5.6%)	0	0
I	1 (5.6%)	1 (11.1%)	0
II	0	2 (22.2%)	1 (14.3%)
III	10 (55.6%)	3 (33.3%)	1 (14.3%)
IV	6 (33.3%)	3 (33.3%)	1 (14.3%)
V	0	0	4 (57.1%)

Data represent the mean and (SD). For Braak stage the number of cases and the % in each group (NCI, MCI or AD)

### Immunohistochemistry

Paraformaldehyde-fixed, paraffin-embedded 4 μm thick olfactory bulb and hippocampal formation tissue sections were deparaffinized, hydrated and treated with Tris-EDTA antigen retrieval buffer (10 mmol/LTris Base, 1 mmol/L ethylenediaminetetraacetic acid (EDTA), 0.05% Tween 20, pH 9) for 20 min at 97 °C in the Pascal Dako Cytomation apparatus. The Dako Autostainer Plus automated slide processor and the EnVision Flex system (Dako, Burlington, ON, Canada) were used to perform all the immunostaining. Brain cases with no pathology were added as negative control, and previously identified immunopositive AD hippocampus or cortical tissue sections added as positive controls for each immunostaining. The following antisera or antibodies were used: neoepitope antisera p20Casp6 10,630 antiserum (1/2000) recognizing the processed active p20 subunit of Casp6 and 10,635 Tau∆Casp6 (1/25,000) produced in our laboratory [27], F25276 antiserum (1/1000) raised against Aβ_1-40_ peptide [36] and PHF-1 antibody (1/5000) against phosphorylated Tau kindly provided by Dr Peter Davies (Department of Neuroscience, Albert Einstein College of Medicine, New York, NY). Slides were counterstained with hematoxylin, dehydrated and coverslipped with Permount mounting medium (Fisher Scientific, Ottawa, ON, Canada).

The neuronal density of AON and ERC tissue was evaluated in three AD cases where both AON and ERC were available, by counting the neurons within a defined area in the counterstained hematoxylin/eosin Casp6 immunostained tissue sections. Neurons were recognized according to their morphological characteristics as defined [24]. At least 500 neuronal nuclei were counted per 3-4 different regions of interest for each tissue section.

### Cresyl violet staining (Nissl staining)

Olfactory bulb sections were deparaffinized with xylene, hydrated in descending series of ethanol concentrations and treated with a 0.1% cresyl violet in water (Sigma-Aldrich, St Louis, MO, USA) solution for 15 min followed by a few seconds of differentiation in 95% ethanol. After staining, the sections were dehydrated in absolute ethanol, treated with xylene and coverslipped using Permount mounting medium (Fisher Scientific, Ottawa, ON, Canada).

### Quantitation of the immunopositive regions of the olfactory bulb

Stained tissue sections were scanned digitally with the MIRAX Scanner (Zeiss, Oberkochen, Germany). Consecutive cresyl violet stained olfactory bulb tissue sections were used to delineate the AON, and the identification of the AON was determined by a neuropathologist (SA) as described [51]. The AON was defined as a group of large pyramidal neurons slightly stained with cresyl violet. The *Atlas of the Human Brain* was used as a reference to identify specific brain regions [40]. Five regions of interest at least were photographed at magnification × 20 with the MIRAX Viewer Program (Zeiss, Oberkochen, Germany). Immunostaining density was obtained with the ImageJ software (NIH, Bethesda, MD, USA) and results were expressed as μm^2^ immunopositive staining/mm^2^ of tissue.

### Statistical analyses

Descriptive statistics are reported for the variables of interest. For categorical variables, we report counts and percentages. For continuous variables whose distribution of values showed evidence of being Normal, we report mean and standard deviation (SD); otherwise we report median and inter-quartile range (IQR). Spearman’s correlations were run to determine monotonic relationship between the different pathologies present in the AON or the ERC and the relationship between the presence in AON and ERC for each marker. The distributions of values for active Casp6, Tau∆Casp6, PHF-1 and Aβ between NCI, MCI and AD were compared with the Kruskal-Wallis (KW) test. When warranted by a conclusion of significant differences from the KW test, post-hoc pairwise comparisons were performed with Dunn’s multiple comparisons test [20, 21]. These comparisons were done separately for the AON and the ERC. Multiple linear regressions were performed to investigate the influence of pathological characteristics present in AON or ERC (these analyses were done separately for the AON and the ERC) on cognitive scores measured. The models were adjusted for sex, age, PMI and years of education. All statistical tests of hypothesis were two-sided and carried out at the level of significance of 0.05. The multiple linear regressions were done using the R software (R Core Team (2016). R: A language and environment for statistical computing. R Foundation for Statistical Computing, Vienna, Austria. URL https://www.R-project.org/. All other statistical analyses were performed using SAS, version 9.3 (SAS Institute, Cary, NC, USA).

## Results

### The AON is the region of the olfactory bulb most immunopositive to active Casp6, Tau∆Casp6, PHF-1 Tau and Aβ

**Fig. 1:**
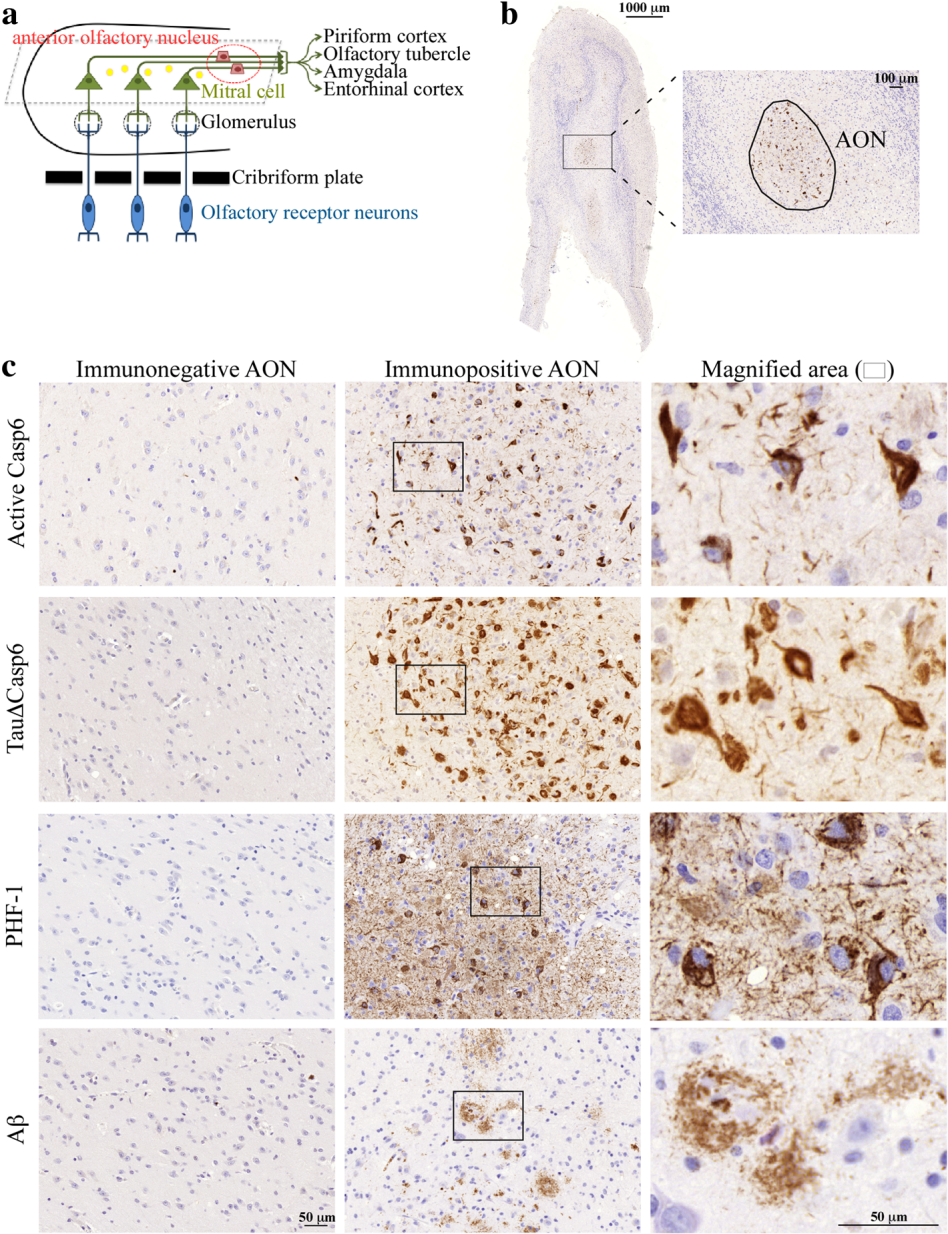
Casp6, Tau and Aβ pathologies are present in the AON of Olfactory Bulb from ROS study subjects. **a** Schematic diagram of the human olfactory bulb and its cell composition (adapted from Saiz-Sanchez D. et al., [49]). **b** Representative image of horizontal section of human olfactory bulb (OB) (bar scale = 1000 μm) stained for active Casp6 with higher magnification showing delineation of the AON (bar scale = 100 μm). **c** Representative images of negative and positive immunohistochemical staining of active Casp6, TauΔCasp6, PHF-1 and Aβ in the AON (bar = 50 μm). The square in the immunopositive AON panel has been magnified 4.5 fold

The AON area of the olfactory bulb whose neurites extend to the ERC (Fig. 1a) was chosen for quantification of Casp6 activity since the AON represents the area that is most strongly immunopositive to the neoepitope antisera against the p20 active subunit of Casp6 and Tau∆Casp6, phosphorylated Tau PHF-1 antibodies and anti-Aβ antiserum (Fig. 1b & c). Strong anti-active Casp6 immunostaining was detected in neurons resembling pre-NFT and mature NFT, and in neuropil threads, as observed previously in AD cortex [27]. Tau∆Casp6 immunoreactivity was detected in NFT-containing neurons and neuropil threads of adjacent tissue sections, and was more abundant than the immunoreactivity of the anti-active Casp6 as expected since this antiserum is considerably stronger and Tau is more abundant in neurons than the Casp6 enzyme. Casp6 activity was not associated with apoptotic neuronal cell death based on the normal appearance of the nuclei of immunopositive neurons. PHF-1 immunostained NFT, although more sparsely than the Tau∆Casp6 antiserum. Conversely, PHF-1 immunostained neuropil threads more abundantly than anti-active Casp6 and Tau∆Casp6 neoepitope antisera. Anti-Aβ immunostained β-amyloid deposits resembling classical β-amyloid plaques and some smaller diffuse deposits. Together, these results show that both the processed form of active Casp6 and Tau∆Casp6, that define Casp6 activity, co-exist with classical AD pathology in the AON of the olfactory bulb.

### Levels of active Casp6 correlate with Tau∆Casp6 and PHF-1, but not with Aβ, in the AON of the olfactory bulb

**Fig. 2:**
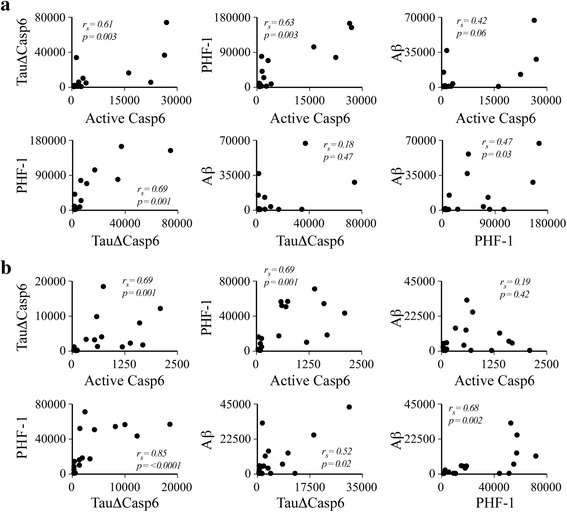
Active Casp6 and TauΔCasp6 staining shows a positive monotonic relationship with PHF-1 staining in AON and ERC. Scatterplots and Spearman’s correlation (r_s_) between the active Casp6, TauΔCasp6, PHF-1 and Aβ immunostaining (μm^2^ staining/mm^2^ of tissue) in the AON (**a**) and in the ERC (**b**)

Spearman’s correlation showed a strong positive relationship between the anti-active Casp6 and the TauΔCasp6 in the AON of the olfactory bulb (*r*_*s*_ = 0.61, *n* = 21, *p* = 0.003) (Fig. 2a). Both the anti-active Casp6 and the Tau∆Casp6 immunostaining showed a strong positive relationship with PHF-1 immunostaining (*r*_*s*_ = 0.63, *n* = 20, *p* = 0.003 and *r*_*s*_ = 0.69, *n* = 20, *p* = 0.001, respectively) indicating, as observed previously in hippocampus [47], an association between Tau NFT pathology and Casp6 activity. By contrast, neither the anti-active Casp6 nor TauΔCasp6 immunostaining showed a relationship with Aβ immunostaining (*r*_*s*_ = 0.44, *n* = 19, *p* = 0.06 and *r*_*s*_ = 0.18, *n* = 22, *p* = 0.47, respectively). The level of PHF-1 showed a weak positive relationship with Aβ immunostaining in the AON (*r*_*s*_ = 0.47, *n* = 22, *p* = 0.03).

Spearman’s correlation also showed a strong positive relationship between anti-active Casp6 and Tau∆Casp6 immunopositivity in the ERC (*r*_*s*_ = 0.69, *n* = 21, *p* = 0.001) (Fig. 2b). As observed in the AON, both the anti-active Casp6 and the Tau∆Casp6 immunostaining showed a strong positive relationship with the PHF-1 immunostaining in the ERC (*r*_*s*_ = 0.69, *n* = 18, *p* = 0.001 and *r*_*s*_ = 0.85, *n* = 19, *p* < 0.0001, respectively). Active Casp6 did not show a relationship with Aβ immunostaining in the ERC (*r*_*s*_ = 0.19, *n* = 19, *p* = 0.42), but Tau∆Casp6 did (*r*_*s*_ = 0.52, *n* = 21, *p* = 0.02). Levels of Aβ immunostaining showed a strong positive relationship with PHF-1 immunostaining only in the ERC (*r*_*s*_ = 0.68, *n* = 19, *p* = 0.002). Together, these results indicate that Casp6 activity is strongly associated with NFT pathology, but not with Aβ pathology, in the AON of the olfactory bulb and in the ERC.

### AON active Casp6 and ERC Tau∆Casp6 increase with AD progression

Table 2 shows descriptive statistics for pathological markers by brain region and clinical diagnosis. A KW test showed statistical significant differences in the levels of active Casp6 in the AON (*χ* ^2^ (2 degrees of freedom) = 6.05, *p* = 0.04). A post-hoc Dunn’s test, showed a difference between AD dementia and NCI, as well as between AD dementia and MCI. By contrast, although Tau∆Casp6 in the AON showed higher amounts in AD dementia as compared to NCI and MCI, the KW test showed that there were no statistically significant differences. This may be explained by the fact that the variability in Tau∆Casp6 values, as measured by the IQR, is greater within AD dementia, NCI and MCI. Similarly for PHF-1 and Aβ, there were no statistically significant differences between the clinical diagnostic groups.

**Table 2:** AON and ERC immunostaining levels in NCI, MCI and AD

Region of interest	Clinical Diagnosis		Active Casp6	TauΔCasp6	PHF-1	Aβ
AON	NCI	Median	837.7	472.9	3655.9	403.3
		IQR	289.0–1481.8	263.0–4182.6	1248.2–23248.5	109.9–658.2
		n	11	11	11	10
	MCI	Median	226.1	980.7	7494.4	452.9
		IQR	118.1–2705.2	458.0–9493.4	651.1–67082.5	136.3–2993.1
		n	6	6	7	7
	AD	Median	23982.4	20400.0	75146.7	27355.0
		IQR	11445.0–26221.2	3087.2–54521.9	41473.2–151857.4	1222.9–55633.7
		n	4	4	5	5
ERC	NCI	Median	69.7	29.3	4138.2	940.7
		IQR	53.8–518.3	10.8–1138.3	615.9–16672.1	106.5–3406.6
		n	8	9	9	9
	MCI	Median	662.4	1035.8	15562.1	96.1
		IQR	14.4–1639.4	246.8–3901.1	9394.8–42729.6	54.2–4787.4
		n	7	7	7	7
	AD	Median	1022.1	7859.5	56051.4	14342.6
		IQR	497.2–1453.1	3181.4–18251.4	53590.7–70360.4	11159.6–24689.1
		n	4	5	3	5

Data represent the median (μm^2^ staining/μm^2^ tissue) and the Inter-Quartile Range (25% percentile-75% percentile). n indicates the number of cases studied with each immunostaining in this region of interest. Comparisons of distributions for pathological markers by clinical diagnosis were done separately in the AON and in the ERC using the Kruskal-Wallis test, followed by a Dunn’s post-hoc test where warranted (results and *p* values are reported in the text)

In the ERC, there were no statistically significant differences in the levels of active Casp6 between NCI, MCI and AD brains. However, differences in levels of ERC Tau∆Casp6 were observed (KW test, *χ* ^2^ (2 df) = 8.37, *p* = 0.01). A post-hoc Dunn’s test, showed a difference between AD and NCI. Similarly, statistically significant differences in the levels of ERC PHF-1 (KW test, *χ* ^2^ (2 df) = 7.13, *p* = 0.02) and Aβ levels (KW test, *χ* ^2^ (2) = 8.32, *p* = 0.01) were obtained and post-hoc Dunn’s test confirmed a difference between AD and NCI, and between AD and MCI. Together, these results indicate higher levels of active Casp6, but not Tau∆Casp6, PHF-1 or Aβ, in the AD AON than in the NCI or MCI AON. By contrast, levels of active Casp6 are not different in NCI, MCI or AD ERC, Tau∆Casp6 is increased in AD ERC relative to NCI ERC levels, but PHF-1 and Aβ levels are increased in AD ERC compared to both NCI and MCI ERC.

### Levels of Casp6 activity in the AON correlate with the levels of Casp6 activity and PHF-1 positive NFT in the ERC

**Fig. 3:**
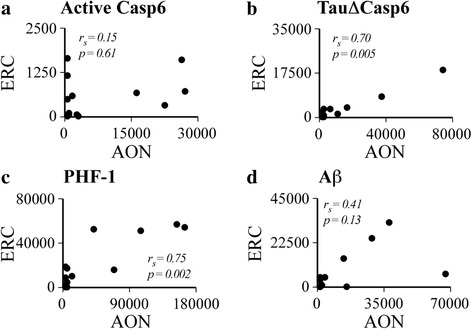
AON TauΔCasp6 and PHF-1 staining shows a positive monotonic relationship with ERC staining. Scatterplots and Spearman’s correlations (r_s_) between the active Casp6 (**a**), TauΔCasp6 (**b**), PHF-1 (**c**) and Aβ (**d**) immunostaining (μm^2^ staining/mm^2^ of tissue) in the AON versus the ERC

**Fig. 4:**
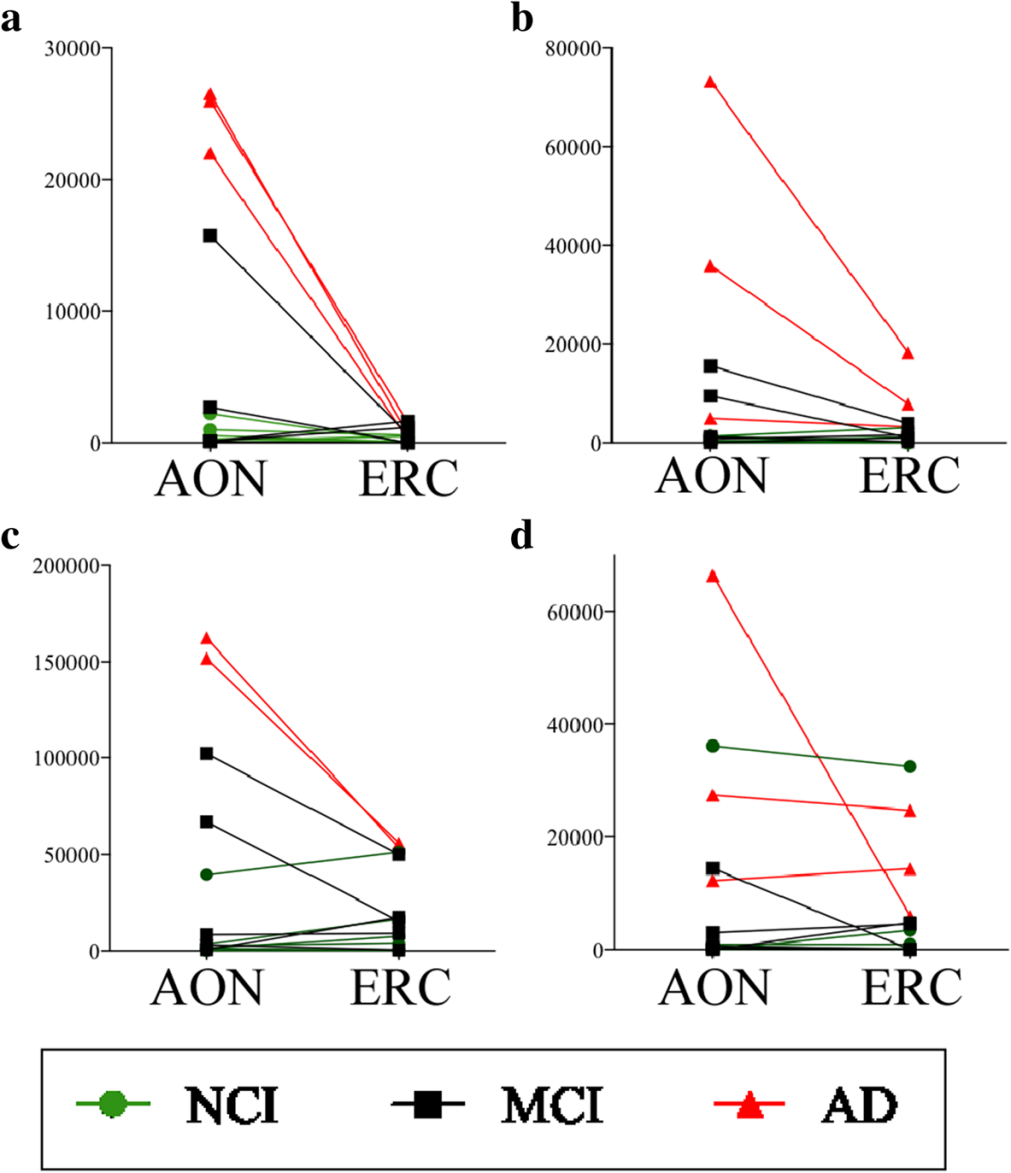
Casp6 and Tau pathologies are more abundant in AON than in the ERC for AD cases. Active Casp6 (**a**), TauΔCasp6 (**b**), PHF-1 (**c**) and Aβ (**d**) immunostaining values (μm^2^ staining/mm^2^ of tissue) in the AON and ERC for 5 NCI, 3 MCI and 3 AD cases where both the AON and ERC were stained from the same individual case

It is expected that if degeneration in the ERC occurs because of the degeneration of input neurons in the AON, then Casp6 activity in the AON should show a relationship with that of the ERC. Spearman’s correlation showed that levels of anti-active Casp6 in the AON did not have a significant relationship with those in the ERC (*r*_*s*_ = 0.15, *n* = 14, *p* = 0.60) (Fig. 3a). However, levels of Tau∆Casp6 immunoreactivity in the AON were associated positively with those in the ERC (*r*_*s*_ = 0.70, *n* = 14, *p* = 0.01) (Fig. 3b). Since cytosolic caspase enzyme levels are lower than the cytoskeletal Tau protein and because Casp6 undergoes rapid turnover by the proteasome [62], we consider Tau∆Casp6 to be a stronger indicator of Casp6 activity than active Casp6. Furthermore, PHF-1 immunostaining in AON were associated positively with that of the ERC (*r*_*s*_ = 0.75, *n* = 14, *p* = 0.002) (Fig. 3c). By contrast, Aβ levels in the AON did not show a relationship with those in the ERC (*r*_*s*_ = 0.41, *n* = 15, *p* = 0.12) (Fig. 3d). These results indicate that degeneration in the AON parallels that of the ERC but does not give any information as to whether degeneration in the AON preceded that in the ERC. To assess this possibility, we compared immunopositive levels where staining were obtained in both the AON and ERC of the same case (Fig. 4). All 3 AD dementia cases and one MCI case showed higher active Casp6 in the AON than in the ERC (Fig. 4a). Two out of 3 AD dementia cases and 2 MCI cases showed higher Tau∆Casp6 in the AON than in the ERC (Fig. 4b). Similarly, PHF-1 levels in AON were higher in AD and 2 MCI cases (Fig. 4c). By contrast, Aβ was higher in AON in only one AD case, other cases showed similar levels.

Together, these results suggest that either the AON is more susceptible to Casp6 activation and PHF-1 immunopositive AD pathology than the ERC or that the AON pathology precedes the ERC pathology and this result in a greater accumulation of Casp6 activity and PHF-1 pathology.

### Higher levels of active Casp6 in the AON correlate with lower cognitive performance

To investigate the association between the pathologies of the AON or ERC and the cognitive performance of individuals, we performed separate regression analyses for five cognitive domains assessed within a year of death. Analyses were done separately for the AON and the ERC. In each regression, we adjusted for sex, age, PMI and years of education. Spearman’s correlation results discussed above showed that active Casp6, TauΔCasp6 and PHF-1 immunostaining were highly associated with one another. Multicollinearity of these three factors was confirmed by their high values of the variance inflation factor found in a preliminary for-diagnosis multiple regression model. To adjust for this multicollinearity, we used a ridge regression approach, which allows for the inclusion of these factors in the same model. The value of the ridge parameter λ was chosen as the one for which the coefficients were relatively stable from the ridge traces. The values selected were λ = 20 and λ = 100 for AON and ERC, respectively. Furthermore, due to the small sample sizes, *p* values for the regression coefficients were computed using a permutation method with 10,000 permutations [17]. All ridge regressions and permutation tests were performed using the package MASS in R [65].

Higher levels of active Casp6 in the AON were associated with lower global cognitive, MMSE, episodic and semantic memory scores (Table 3). Tau∆Casp6 and Aβ levels in the AON were marginally negatively associated with the MMSE score and GCS, respectively. By contrast, PHF-1 levels in the AON were not associated with any of the cognitive measures. In the ERC, active Casp6 and Tau∆Casp6 levels were marginally negatively associated with perceptual speed, and episodic memory or perceptual speed, respectively. Tau∆Casp6 levels were however negatively associated with MMSE scores, a feature observed also for PHF-1, although the association was weak. Aβ levels did not associate with any of the cognitive measures. These results indicate that AON active Casp6 levels are associated with cognitive decline, and interestingly, the association is with episodic and semantic memory, two types of memory function initially altered in AD.

**Table 3:** Ridge regression coefficients of cognitive performance scores from AON and ERC pathologies

	AON (*n* = 19)			ERC (*n* = 18)		
Predicting cognition	Marker	Coefficient	*p* value	Marker	Coefficient	*p* value
GCS	Active Casp6	−0.18	**0.01**	Active Casp6	−0.02	0.40
	TauΔCasp6	−0.08	0.29	TauΔCasp6	−0.05	0.06
	PHF-1	−0.06	0.33	PHF-1	−0.03	0.27
	Aβ	−0.18	**0.04**	Aβ	−0.03	0.27
MMSE	Active Casp6	−1.97	**0.001**	Active Casp6	−0.29	0.23
	TauΔCasp6	−1.36	**0.05**	TauΔCasp6	−0.72	**0.01**
	PHF-1	−0.92	0.06	PHF-1	−0.41	**0.04**
	Aβ	−0.90	0.21	Aβ	−0.30	0.14
Episodic Memory	Active Casp6	−0.31	**0.01**	Active Casp6	−0.02	0.67
	TauΔCasp6	−0.13	0.32	TauΔCasp6	−0.07	0.07
	PHF-1	−0.14	0.17	PHF-1	−0.05	0.24
	Aβ	−0.28	0.05	Aβ	−0.05	0.27
Working memory	Active Casp6	−0.06	0.26	Active Casp6	−0.02	0.21
	TauΔCasp6	−0.03	0.59	TauΔCasp6	−0.01	0.64
	PHF-1	−0.004	0.93	PHF-1	−0.004	0.81
	Aβ	−0.09	0.16	Aβ	0.001	0.95
Semantic memory	Active Casp6	−0.15	**0.03**	Active Casp6	−0.01	0.69
	TauΔCasp6	−0.11	0.14	TauΔCasp6	−0.05	**0.05**
	PHF-1	−0.07	0.25	PHF-1	−0.02	0.55
	Aβ	−0.16	0.06	Aβ	−0.03	0.28
VSA	Active Casp6	−0.09	0.12	Active Casp6	−0.01	0.62
	TauΔCasp6	−0.01	0.91	TauΔCasp6	−0.01	0.84
	PHF-1	0.02	0.72	PHF-1	−0.01	0.57
	Aβ	−0.06	0.41	Aβ	−0.03	0.21
PS	Active Casp6	−0.08	0.23	Active Casp6	−0.05	**0.05**
	TauΔCasp6	−0.07	0.32	TauΔCasp6	−0.05	**0.04**
	PHF-1	−0.02	0.71	PHF-1	−0.02	0.29
	Aβ	−0.04	0.64	Aβ	−0.02	0.31

Data represent the ridge regression analysis of the different markers in the AON and the ERC and the cognitive scores. Model adjusted for sex, age, PMI and years of education, in bold *p* values less or equal to 0.05

## Discussion

In this study, the levels of Casp6 activity were assessed in the olfactory bulb of NCI, MCI and AD dementia individuals to determine if previously observed Casp6 activity in the ERC was associated with degeneration in the olfactory bulb since olfactory bulb neurons project their axons to the ERC. The results of the study revealed that (1) Casp6 activity is highest in and mostly restricted to the AON of the olfactory bulb and co-exists with Tau, but not Aβ, AD pathology, (2) Casp6 activity increases in the AON with the progression of disease and appears to be higher than that of Casp6 activity in the ERC, and (3) active Casp6 levels in the AON are negatively associated with cognitive performance.

The co-existence of Casp6 activity with Tau pathology in the AON of the olfactory bulb is consistent with our previous observations of an intimate relationship between Casp6 activity and PHF-1 Tau pathology in the ERC and CA1 regions of the hippocampus [47]. The fact that both Tau and Casp6 are localized in the neuronal cytosol and are involved in axonal degeneration suggests that they could both be part of a neurodegeneration pathway in AD neurons [18, 45, 55]. Casp6 cleavage of Tau at amino acid 402 [27] may impact Tau’s polymerization, as observed for cleaved Tau at amino acids 391 and 421 [1, 10]. Cleavage of Tau may also alter Tau’s function in the stabilization of microtubules, in a manner similar to the phosphorylation of Tau at amino acid 396 [13]. Whether NFT formation and Casp6 activity occur as parallel pathways of degeneration or as part of the same pathway remains to be determined, and will be an important question to resolve eventually as the answer would have a significant implication in the development of treatments against neurodegeneration in AD.

In contrast to its association with NFT pathology, Casp6 activity in the AON is not associated with Aβ extracellular deposits. The lack of association between Casp6 activity and Aβ is consistent with our previous observations in the hippocampus [3, 27, 47]. Our results differ from several reports indicating β-amyloid toxicity associated with caspase activation [7, 14, 15]. Nevertheless, our analyses cannot exclude the possibility of toxic Aβ oligomers triggering neuronal Casp6 activation. It is also important to consider that Aβ production can be a consequence of Casp6 activation in primary cultures of human CNS neurons [32, 36, 38], a fact that would be consistent with Casp6 activity preceding Aβ over-production and deposition as extracellular plaques.

This study shows that Casp6 activity in the AON could be added as an additional biomarker of neurodegeneration associated with AD. First, the AON active Casp6 levels were significantly higher in AD compared to AON NCI or MCI, indicating increased levels of enzyme with progression of disease. A similar trend was observed with Tau∆Casp6 but did not reach statistical significance because of the large variability amongst samples. Second, the levels of Tau∆Casp6 in the AON were positively and strongly associated with the levels of Tau∆Casp6 in the ERC. Similar results were observed for PHF-1 pathology. Third, the higher levels of Casp6 activity, determined by both the presence of active Casp6 and Tau∆Casp6, and PHF-1 Tau, in the AON than in the ERC of individual MCI and AD cases suggest that degeneration in the AON could precede degeneration in the ERC. However, our approach cannot exclude that ERC degeneration leads to AON degeneration or that the two pathologies occur concomitantly. More intensive studies will be required to determine which of these three possibilities explains the relationship between AON and ERC degeneration. The number of available cases evaluated limited our study. Furthermore, a longitudinal study would be required to determine if pathology in one area of the brain precedes another. This longitudinal study would require the ability to observe the degeneration in live patients. This is likely to soon become possible with the evolution of PET ligands against tangles and Aβ, but is not yet possible for Casp6 activity. Furthermore, as proposed by Attems [4–6], olfactory biopsies could be done to assess the diagnostic potential of the olfactory bulb, and to evaluate the efficiency of treatments against AD.

This study shows an association between higher levels of AON active Casp6 and lower memory performance. Indeed, higher levels of Casp6 associate with lower global cognitive, MMSE and episodic and semantic memory, scores. These results are consistent with the association of impaired odor identification and lower global cognition and a more rapid rate of episodic and semantic memory decline in MCI [68]. The fact that episodic and semantic memory impairment occurs before AD dementia [43] suggests the involvement of Casp6 activity at a very early stage of the disease. In other studies, an association between olfactory bulb and tract atrophy and MMSE scores was observed [60, 61]. Given the role of Casp6 in axonal degeneration [45, 55], it would be of interest to determine eventually if Casp6 activity may be responsible for the atrophy of the olfactory bulb and tract. Surprisingly, AON Tau∆Casp6 showed only a marginal association with a decline in MMSE score and no association with the other cognitive measures. Similarly, no association was observed between AON PHF-1 levels and cognitive measures. These results suggest that the presence of Tau pathology in the AON is not associated with cognitive performance. Similarly, the AON levels of Aβ associated very weakly with lower GCS and not with any of the other measures of cognition. However, with larger numbers, significance may be achieved. For example, an association between olfactory Tau pathology and clinical dementia in AD and MCI subjects has been reported [4, 5].

In the ERC, Tau∆Casp6 and PHF-1 levels associated negatively with the MMSE score. Active Casp6 and Tau∆Casp6 levels both associated negatively but very weakly with perceptual speed performance. Similarly, a very weak association between Tau∆Casp6 and semantic memory was also observed. Aβ levels did not associate with any of the cognitive measures. Previously, we did not observe an association between cognition and Casp6 activity in the ERC when combining NCI, MCI and AD groups, but detected a significant association between episodic and semantic memory and Casp6 activity in the NCI subjects ERC [3, 47]. Larger numbers in each subgroup will be required for a solid conclusion to be drawn. Since the focus of this study was the olfactory bulb, we did not pursue the study in the ERC.

## Conclusions

In conclusion, these data support the possibility that the Casp6 activity in the AON of the olfactory bulb reflects degeneration in the ERC and suggest that Casp6 activity in the olfactory bulb could represent degeneration associated with cognitive decline and early AD. Furthermore, Casp6 in the AON could be used as an early marker to detect the initiation of pathways of degeneration in AD.

## Abbreviations

AD: Alzheimer disease
AON: Anterior olfactory nucleus
APP: Amyloid precursor protein
CA1: Cornus ammonis 1
Casp3: Caspase-3
Casp6: Caspase-6
Casp7: Caspase-7
ERC: Entorhinal cortex
IQR: Inter-quartile range
MCI: Mild cognitively impaired
MMSE: Mini-mental state examination
NCI: Non-cognitively impaired
NFT: Neurofibrillary tangles
PHF-1: Paired helical filament
PMI: Post-mortem interval
ROS: Religious order study
SD: Standard deviation
